# Longitudinal Study of the Bulk Tank Milk Microbiota Reveals Major Temporal Shifts in Composition

**DOI:** 10.3389/fmicb.2021.616429

**Published:** 2021-02-23

**Authors:** Davide Porcellato, Marit Smistad, Alberto Bombelli, Ahmed Abdelghani, Hannah Joan Jørgensen, Siv B. Skeie

**Affiliations:** ^1^Faculty of Chemistry, Biotechnology and Food Science, Norwegian University of Life Sciences, NMBU, Ås, Norway; ^2^Norwegian Veterinary Institute, Oslo, Norway; ^3^TINE SA, Oslo, Norway; ^4^Department of Agrotechnology and Food Science, Wageningen University and Research, Wageningen, Netherlands

**Keywords:** raw milk microbiota, bulk tank, sequence variants, longitudinal study, milk quality

## Abstract

Introduction of microbial contaminations in the dairy value chain starts at the farm level and the initial microbial composition may severely affect the production of high-quality dairy products. Therefore, understanding the farm-to-farm variation and longitudinal shifts in the composition of the bulk tank milk microbiota is fundamental to increase the quality and reduce the spoilage and waste of milk and dairy products. In this study, we performed a double experiment to study long- and short-term longitudinal shifts in microbial composition using 16S rRNA gene amplicon sequencing. We analyzed milk from 37 farms, that had also been investigated two years earlier, to understand the stability and overall microbial changes over a longer time span. In addition, we sampled bulk tank milk from five farms every 1–2 weeks for up to 7 months to observe short-term changes in microbial composition. We demonstrated that a persistent and farm-specific microbiota is found in bulk tank milk and that changes in composition within the same farm are mostly driven by bacterial genera associated with mastitis (e.g., *Staphylococcus* and *Streptococcus*). On a long-term, we detected that major shift in milk microbiota were not correlated with farm settings, such as milking system, number of cows and quality of the milk but other factors, such as weather and feeding, may have had a greater impact on the main shifts in composition of the bulk tank milk microbiota. Our results provide new information regarding the ecology of raw milk microbiota at the farm level.

## Introduction

Production of high-quality dairy products requires a high-quality raw milk. The initial microbial quality of raw milk is one of the most important parameters used by the dairy industry to assess the milk prior to production. It is important for the dairy industry to avoid the introduction in the dairy chain of spoilage microbes that can cause quality-deterioration of milk products. In particular, presence of spore-forming microorganisms, and thermoduric enzymes produced by gram-negative psychrotrophs, which are not destroyed during pasteurization, confer significant quality problems in milk and dairy products (Mateos et al., 2015). Knowing and understanding the composition and temporal changes, as well as the potential causes of these changes in the community structure of raw milk is therefore important for the dairy industry. This can inform action to reduce spoilage and limit economical costs and food waste caused by quality problems.

The microbiota of raw milk originates from multiple sources of contamination. While the initial microbial load in milk from the healthy udder is low, there is a continuous increase of microorganisms in the milk as it flows from the cow to the bulk tank. With contaminations introduced from the teat apex, the milking system and farm environment, the final composition in a milk microbiota of the bulk tank milk is highly diverse (as reviewed by Parente et al., 2020). Furthermore, milk from cows with undetected clinical mastitis or subclinical mastitis, may contain a high number of the causal pathogen, but may still be collected and delivered for production. Other factors such as farm management practices and seasonality have also been shown to significantly impact the microbiota detected in raw milk (Elmoslemany et al., 2010). Despite the high complexity of the raw milk microbiota and the diversity detected between farms, Parente et al. (2020) identified a core microbiota from milk samples collected in several countries. The most abundant taxa found in the raw milk core microbiota included psychrotrophs (e.g., *Pseudomonadaceae* and *Aeromonadaceae*), gut or rumen (e.g., *Lactobacillaceae*, *Clostridiaceae*, and *Ruminococcaceae*) and teat skin bacteria (e.g., *Corynebacteriaceae* and *Corynebacteriaceae*). Psychrotrophic bacteria are usually not found in the udder or teat microbiota, but they are a result of the environmental contamination from equipment and bacterial growth in milk during storage in the bulk tank.

While a high diversity of bulk tank milk microbiota has been described in several studies, the stability of the microbiota within the same farm and its longitudinal changes has been investigated to a lesser degree. The Norwegian dairy industry has a long tradition for animal recordings and in 2019, 97% of the dairy herds were included in the Norwegian Dairy Herd Recording System (NDHRS; TINE Rådgivingog Medlem, 2019). This database includes information on parameters such as barn type, herd size and milking system, but also more dynamic recordings such as feeding, bulk milk somatic cell count (SCC) and mastitis treatments. Previously, we showed that bulk tank microbiota differs between farms, geographical regions and sampling times (Skeie et al., 2019). In that study, milk samples were collected during the winter months in 2017, following a summer season with average temperatures and rainfall (Skeie et al., 2019). The next sampling, during the winter months of 2019, followed a drought summer (2018) which led to adjustments in the quality of feed and the amounts of concentrate given.

To better understand the causes of diversity and fluctuations of bulk milk microbiota composition, we investigated long and short-term longitudinal shifts in bulk tank microbiota to identify factors that might impact its composition. By resampling the same farms in the same season after two years, we aimed to determine if geographical and intra-farm differences were still present and identify farm factors that might affect changes in microbiota.

## Materials and Methods

### Selection of Farms, Milk Sampling, and Bacterial Count

Two investigations of microbiota in bulk milk were carried out. In the first investigation, the microbiota of bulk tank milk samples from 37 farms was analyzed with a 2-year interval. The first sample set was collected between January and March 2017 and the microbiota of these samples was described previously (Skeie et al., 2019). The second sample set for repeated analyses was collected between January and March 2019. In this study, samples were collected from each farm three times every 2–3 weeks and the microbiota of the 2019 samples was compared to results from the study in 2017 to evaluate long term changes in composition. These 37 farms deliver bovine raw milk to two regional dairy plants (area A and B) situated 110 km apart in the southern part of Norway. Nineteen farms were from area A and 18 from area B.

In the second investigation, five farms were selected for a longitudinal study of bulk milk microbiota, and samples were collected in the period February–August 2019. Three (L1–L3) of the five farms were sampled in the period February–August 2019 and two farms (L4–L5) were sampled between April–May and August–September. Bulk milk was collected from these five farms at an interval of 1–2 weeks for the entire sampling period.

The milk sampling for both investigations was performed as previously described (Skeie et al., 2019). Briefly, raw milk was collected during the transfer from the bulk tank to the tanker truck in a sterile 50 mL tube using an automatic procedure ensuring. The sampling procedure is automatic and ensures representative bulk milk samples. Milk was stored at 4°C and transported chilled to the dairy plant. For only the samples collected in the first investigation (samples from 2017 and 2019), an aliquot (1 mL) was taken to perform the total bacterial count (TBC) by flow cytometric counting of the bacteria using the Bactocount IBCm instrument (Bentley Instrument Inc., Chaska, MN, United states) according to manufacturer’s instructions (Bentley Instrument Inc.). The remaining milk was sent frozen to the Faculty of Chemistry, Biotechnology and Food Science laboratory, NMBU, for DNA extraction and sequencing analysis.

### Data Collection From Farms and Meteorological Data

For the first study, data on herd size, barn type, milking system and milk quality parameters, bulk milk SCC and TBC, were extracted from the NDHRS. SCC and TBC data are obtained twice a month from samples for each farm.

Meteorological data (average temperature and precipitation) from two central weather stations in area A and B were obtained from the Norwegian Meteorological Institute’s database (Norsk Klimaservicesenter, 2019) for February 2017 and 2019 and for the summer months (May–August) of 2016 and 2018 to reflect period of roughage production.

In addition to the data collected from the NDHRS, an online questionnaire was sent out to all the 37 farms included in the study. Several questions regarding roughage/silage production reduction (in %), amount of dry matter in feed, acquisition of feed and import of feed from outside Norway were included in the questionnaire (Supplementary Table 2). The regular growing season in the two areas of interest for this study perform three cuts of grass during the summer season.

The five farms included in the second study were all free stalls with between 12 and 50 dairy cows. The farmers were interviewed regarding events during the sampling period, e.g., the start/end of pasture season, purchase of animals, mastitis treatments and procedures for cleaning of the milking machine. One of the farms (L2) was organic while the remaining four were conventional.

### DNA Extraction and 16S rRNA Amplicon Sequencing

Bacteria isolation from the milk was performed from 40 mL of milk by centrifugation for 10 min at 8,000 × *g*, and the pellet was resuspended and washed twice with 1 mL of 2% sodium citrate water (w/v). DNA was extracted according to the methods described by Skeie et al. (2019) and stored at −20°C until analysis. Library preparation and sequencing was performed as described before (Skeie et al., 2019). The V3 and V4 regions of the bacterial 16S rRNA were amplified using the method described by Porcellato and Skeie (2016) with minor changes. Two μL of DNA was added to the PCR reaction mix containing 1× HF Buffer (Bio-Rad, Hercules, CA, United states), 0.2 mM of each primer, 200 mM of each dNTPs and 0.02 U/mL of iProofTM *taq* polymerase (Bio-Rad). The PCR amplification conditions and sequencing protocol was similar to those previously reported (Porcellato and Skeie, 2016). The raw data for both experiments have been deposited at the European Nucleotide Archive with accession number PRJEB39376. Sequences from the first sampling of the milk from 37 farms were deposited at the European Nucleotide Archive with accession number PRJEB24669. Reads were quality filtered and trimmed using the DADA2 package (Callahan et al., 2016) using truncating of forward reads set to 265 bases and truncating of reverse reads set to 220 bases. The error model in DADA2 was created using 1 million random filtered reads. Sequence variants (SVs) was inferred using the DADA2 algorithm and removal of chimeras was performed using the function “removeBimeraDenovo” in the DADA2 R package (Callahan et al., 2016). Sequence variants shorter than 375 base pairs were removed from the final table. Taxonomy was assigned using the Decipher R package against the SILVA SSU database (Quast et al., 2013; Wright, 2015).

### Statistical Analysis

Statistical analyses were performed using R, version 3.3.3 (R Core Team, 2017). The SV table was normalized using the cumulative-sum scaling method in the R package “metagenomeSeq” (Paulson et al., 2013). Alpha and beta diversity analysis were performed using the R package vegan (Oksanen et al., 2017). Chao1 estimate and Shannon diversity were used to calculate the alpha diversity and comparison of the alpha diversity indexes between group levels was performed using the Kruskal–Wallis test and the Wilcoxon rank-sum test. Multivariate homogeneity of group dispersion was calculated using the function “betadisper” available in the R package vegan. Permutational analysis using dissimilarity matrix (“adonis” function from the R package “vegan”) was used to test differences in the composition of the community between groups of samples (number of permutation 999). Bray–Curtis dissimilarity matrixes were selected as input for ordination analysis using non-metric multidimensional scaling (NMDS). Differential abundance between groups of samples was computed with analysis of compositions of microbiomes with bias correction (Lin and Das Peddada, 2020). Samples with less than 2,000 sequences were not considered for statistical analysis, but they were included in the relative and absolute abundance analysis. The TBC was calculated per mL of milk and then transformed to log TBC/mL. For samples in the first experiment, the relative abundance of each taxon was (1) corrected for the average number of ribosomal RNA operons obtained for each genus from the rrnDB database (Stoddard et al., 2015) and (2) transformed to absolute value (TBC/mL) after computing the percentage of that taxon according to the total levels of bacteria obtained for each sample. We performed linear mixed modelling (LMM) using the lmer function from the lme4 package for alpha diversity indexes (Chao1 and Shannon). Different strategies of data transformation were used to assure the normality of the dependent variable (checked with Shapiro–Wilk test of normality in R). Sampling periods, geographical area, milk quality and other farm parameters were included as independent variables. Farm was included as a random intercept to control for repeated measurements design.

## Results

### Long-Term Changes in Bulk Tank Microbiota

In total, 37 farms were included in the study to evaluate shifts in microbiota between samplings of two years apart. Data on average temperature and precipitation were obtained for both years and the previous summer. Overall, farms in area A had a larger average herd size and a higher proportion of automatic milking systems (AMS) compared to farms in area B (Table 1). Between 2017 and 2019, three farms switched from tie stall pipeline milking system (TSP) to AMS in area A while no such changes were registered in area B.

**TABLE 1:** Descriptive data of the farms included in the study grouped by geographical area and year of sampling.

Dairy/year	A 2017	A 2019	B 2017	B 2019
Number of farms	19	19	18	18
Number of farms, type, and milking system				
Free stall automatic (AMS)	9	12	6	6
Free stall parlor (FSP)	3	3	6	6
Tie stall pipeline (TSP)	7	4	6	6
Mean number of cows (standard deviation, SD)	41.1 (20.5)	40.4 (23.6)	33.9 (13.2)	32 (12.1)
Number of small farms^1^	10	7	8	9
Number of large farms^1^	9	12	10	9
Mean (SD) average somatic cell count (SCC) (in 1,000)	140.6 (52.2)	129.3 (47.4)	117.8 (39.6)	115.1 (52.3)
Mean (SD) average bacterial load (TBC)^2^	4.2 (0.3)	4.4 (0.3)	4.1 (0.3)	4.2 (0.4)
Average milk production per cow per year^3^	8064 (927)	8415 (1233)	7739 (726)	8268 (1314)
Mastitis treatments, mean (SD)^4^	0.2 (0.2)	0.2 (0.1)	0.1 (0.1)	0.1 (0.2)
Prevalence of SCC > 200.000, mean (SD)^5^	22.4 (11.2)	26.7 (11.3)	21.2 (6.2)	19.5 (7.3)
Amount of concentrate, Mean (SD)^6^	24.3 (5.1)	26.1 (5.8)	31.7 (5.7)	30.6 (9.9)
Average temperature (May–August, in°C)^7^	15.3	17.6	15.9	18.2
Average precipitation (May–August, in mm)^7^	80.8	39.7	120.1	45.0
Average Temperature (January–March, in °C)	−3	−1	−1.9	−0.1
Average precipitation (January–March, in mm)	40.3	56.5	46.9	72.7
Collection trucks^8^	3	3	1	1

*^1^Farm sizes were divided in small and large if the number of cows were over the Norwegian average farm size (28 cows). ^2^Data in logarithm. ^3^Milk production per cow/year (kg energy corrected milk). ^4^Number of mastitis treatments per year, divided by number of cows. ^5^Percent of the cows with SCC > 200.000 on individual test days during the last year. ^6^Kg concentrate used per 100 kg produced milk (Energy corrected milk). ^7^Data from harvesting season previous summer 2016 and 2018, respectively. ^8^Number of trucks that collected the milk during each sampling.*

The average bulk SCC and the proportion of cows with subclinical mastitis (>200.000 cells mL^–1^) were higher in farms in area A. The farms in area A used less concentrate compared to area B. Meteorological data showed that area A had a lower average temperature in both years of sampling, but the average precipitation was higher in year 2019 compared to 2017 in both areas. Meteorological data showed that the summer before the 2019 sampling were quite dry and warm, whereas the summer before the 2017 sampling were more normal for a Norwegian summer.

Thirty-three out of 37 farms from which samples were collected also in the first study also completed the questionnaire. A reduction in crop was detected in all the three cuts performed in the summer 2018 during the drought. The farms reported an average reduction in crop of 42% in the first cut, 71% for the second, and 36% for the third cut (Supplementary Table 2). This reduction severely impacted the feeding regime used for the winter season. Firstly, the reduction in crop production affected the amount of concentrate that was given to the dairy cows in the period of sampling in 2019 compared to the same period in 2017 in 19 of 33 farms (Supplementary Table 2). Secondly, the farmers reported an increase in purchased feed (roughage). Twenty-three farms confirmed that they bought roughage (mainly straw and silage) and seven of these imported roughage from abroad (Iceland, Denmark, and Sweden).

**FIGURE 1:**
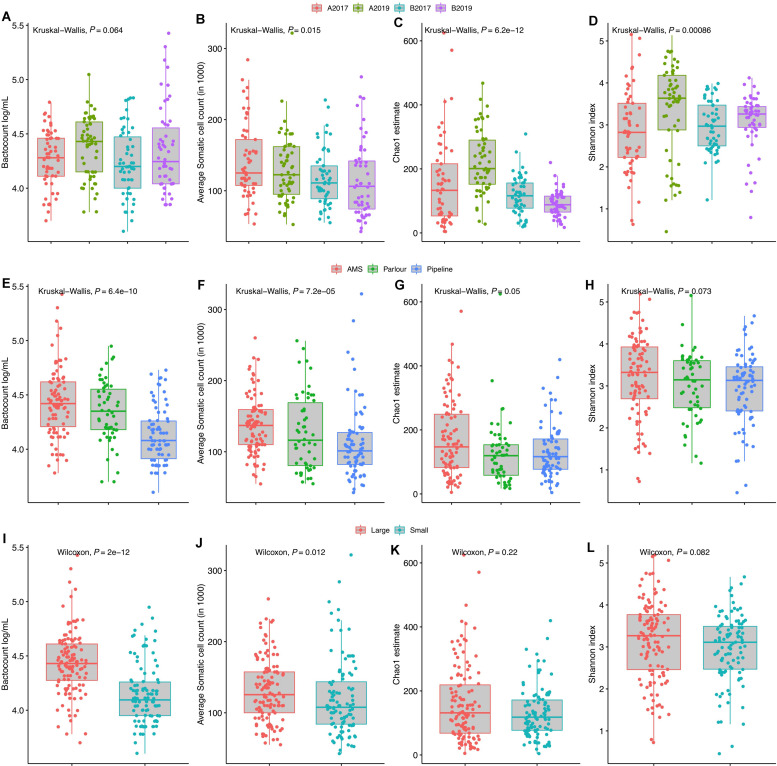
Distribution of total bacterial counts, average somatic cell counts, alpha richness and diversity grouped by year and geographical area **(A–D)**, milking system **(E–H)**, and farm size **(I–L)**. The average number of cows in Norway (28) was used to group the farms into “small” and “large” farms. *P*-value for the non-parametric analysis of variance (Kruskal–Wallis or Wilcoxon test) is reported in each plot.

Somatic cell count and TBC were detected to be lower for the TSP milking system as well as for smaller farms (<28 cows) (Figure 1). These data were correlated as the majority of the farms with a TSP milking system (12 out of 13) were small farms. However, farm size and milking system did not impact the alpha diversity indexes significantly (*P* > 0.05, Figure 1) but a wider distribution of the richness was identified in farm with AMS. The year of sampling was also correlated with TBC in milk, and a higher microbial load was detected in milk samples collected in 2019 compared to 2017 in both areas. The increase in TBC was positively and negatively associated with the richness for the year 2019 in area A and B, respectively.

Two indicators (Chao1 and Shannon index) of alpha diversity within samples were used to study the microbial richness and diversity within the community, respectively. A multivariate linear mixed-effect regression model was used to assess factors associated with alpha diversity. Both richness and diversity were associated with changes in alpha diversity between the years of sampling and the interactions between the year and geographical areas were also significant (*P*-value < 0.05). The TSP milking system was associated with richness but not with diversity, while no associations between the farm size and milk quality (SCC and TBC) with the two alpha diversity indexes were detected (data not shown).

**FIGURE 2:**
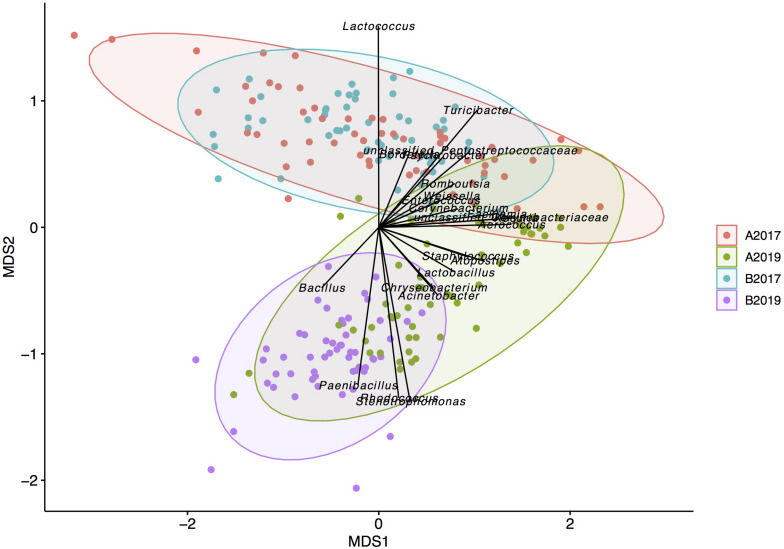
Non-metric multidimensional scaling of the milk microbiota grouped by year and geographical area. Only strong significant taxa (envfit function *P* < 0.001) are reported as predictors onto the ordination.

Bray–Curtis distances were used to study the between-sample diversity. The average SCC was not associated with the microbiota composition (adonis *P*-value = 0.1) while the milking system had a higher *P*-value (0.012) with the microbiota composition compared to year, geographical area and bacterial level (*P*-value ≤ 0.001). Multivariate homogeneity of group dispersion (beta dispersion) showed that milk samples from farms in area B were more similar within the group in both sampling years compared to the samples obtained in area A (Supplementary Figure 1). Correlation analysis of the genera abundance with the first two axis of the NMDS analysis showed that several genera correlated with the beta diversity of the samples (Figure 2). In particular, the genera *Bacillus*, *Paenibacillus*, *Stenotrophomonas*, and *Rhodococcus* were correlated with the milk samples obtained from the year 2019 while *Lactococcus* was highly correlated with samples from 2017. *Pseudomonas*, *Streptococcus*, and *Macrococcus* did not correlate (envfit *P*-value > 0.05) with the beta diversity of the samples indicating that these genera did not influence the between-sample diversity.

**FIGURE 3:**
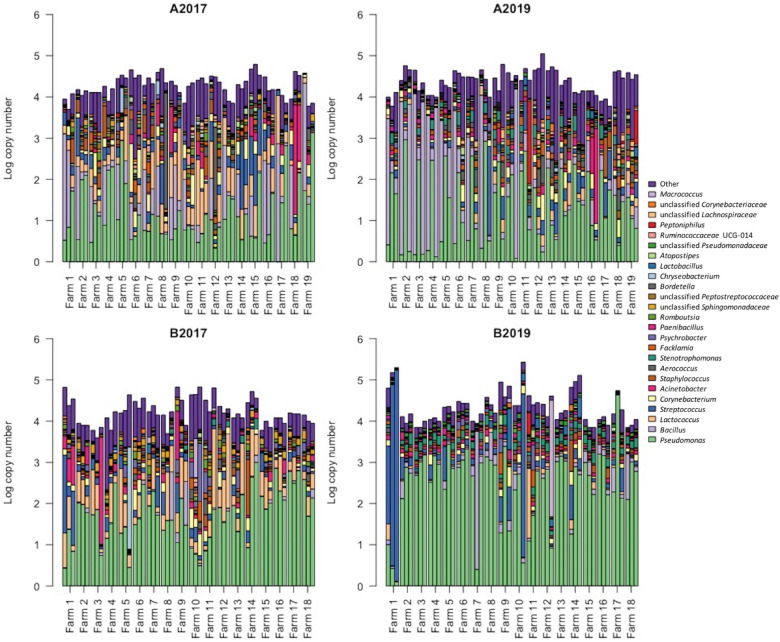
Distribution of the 25 most abundant genera detected in raw milk samples. Each bar represents a sample of milk and each farm was sampled three times. The height of each bar indicates the absolute level of bacteria (in log TBC). The color distribution of each bar was obtained by (1) correcting the relative abundance by the average number of ribosomal RNA operons obtained for each genus and (2) normalizing the relative abundances (in %) against the absolute bacterial level. A: Farm 1–19 collected in area A; B: farm 1–18 collected in area B. 2017: samples collected in the year 2017 and 2019: samples collected in 2019. Data from 2017 were previously published in Skeie et al. (2019).

**FIGURE 4:**
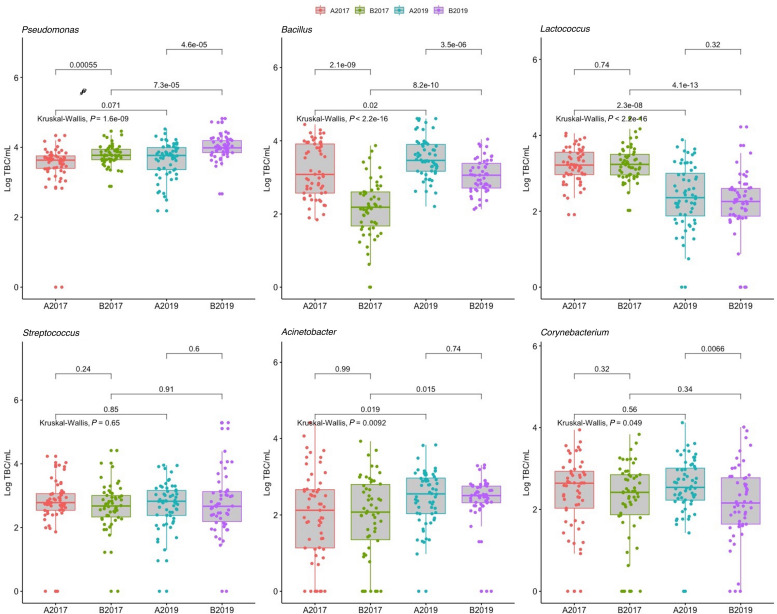
Distribution of total bacterial counts of the six most represented genera of bacteria detected in 222 samples of raw milk from farm bulk tanks grouped by year of sampling and geographical area. Kruskal–Wallis test was used to test significant differences between groups while, pairwise comparison between group of samples was performed using the Wilcoxon test. Data from 2017 were previously published in Skeie et al. (2019).

Between the sampling years, the four most abundant taxa detected in the bulk tank milk (*Pseudomonas*, *Bacillus*, *Lactococcus*, and *Streptococcus*) were similar, with *Pseudomonas* being the dominant genus with 32 and 41% of the reads in the years 2017 and 2019, respectively (Figure 3). The highest amount of *Pseudomonas*, both in absolute value and relative to the microbiota, was detected in milk collected in 2019 from area B, while no significant differences were found between the years for milk samples collected in area A (Figures 3, 4 and Supplementary Figure 2). *Bacillus* significantly increased in abundance in samples collected in 2019 compared to samples collected in 2017 (from 18.6 to 13.4% of the reads and average log TBC/mL of 3.3 and 2.7, Figure 2). *Bacillus* was detected with a relative abundance over 50% of the total reads in 4 and 12 samples from milk collected in geographical area A in 2017 and 2019, respectively, and in only two milk samples from area B in 2019. A significant decrease in *Lactococcus* between the years was observed. In 2017, *Lactococcus* comprised 13% of all the reads compared to 2.3% in 2019 (from log 3.2 to 2.3 TBC/mL, Figure 4) was detected between the years. *Streptococcus* was the only genus, among the four most abundant, that did not significantly change in abundance between the years (average 6.6% and log 2.7 TBC/mL) although milk samples from two farms in area B in 2019 had an increased abundance of *Streptococcus* (>50%) compared to 2017. Farm 1 from area B had an increasing amount of *Streptococcus* over the three samplings in 2019. These three samples also presented a higher bacterial load (log TBC 4.8, 5.2, and 5.3) and a high SCC count (1,62,000, 1,40,000, and 2,60,000 per mL of milk) for the months January, February, and March. The genus *Streptococcus* was also detected in high amounts in the milk collected from the same farm in the previous sampling (2017).

**FIGURE 5:**
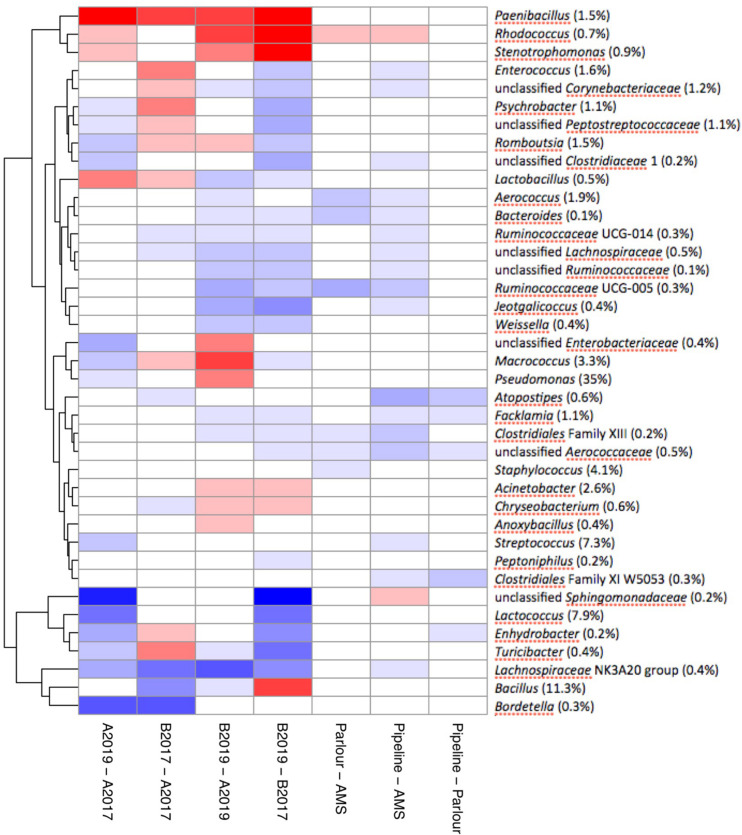
Pairwise differential abundance of the most abundant genera which account for over 95% of the total milk microbiota. Analysis of compositions of microbiomes with bias correction was used to check pairwise association for each genus between the year, geographical area and milking system. Values are reported in log-ratio and colored by differential abundance ratio between the groups.

Differential abundance analysis was used to study genera that were positively associated with different factors. Neither SCC nor mastitis incidence were associated with any of the genera that accounted for more than 95% of the total microbiota (data not shown). The majority of the significant different levels were detected between the year and the geographical area. In particular, the genera *Paenibacillus*, *Rhodococcus*, and *Stenotrophomonas* were significantly correlated with samples collected from area B and samples collected in 2019 (Figure 5). Milk samples from area B collected in 2019 were also significantly enriched with *Pseudomonas* and *Macrococcus* compared to milk samples from area A, while they contained a lower amount of rumen and teat microbiota associated genera (e.g., genera within the families *Ruminococcaceae*, *Aerococcaceae*, *Lachnospiraceae*, and *Corynebacteriaceae*; Figure 5). The level of *Bacillus* remained stable between the years in area A, and was overall detected in higher levels in area A compared to area B although a significant increase was detected in area B from 2017 to 2019. The milking system was associated with the abundance of some taxa. The highest number of significantly different genera between the milking system was detected between TSP and AMS. Most of the genera enriched in AMS samples are commonly found in rumen and teat microbiota, such as *Facklamia*, *Ruminococcaceae*, *Aerococcus*, and *Corynebacteriaceae* (Figure 5).

### Longitudinal Changes in Bulk Tank Microbiota

**FIGURE 6:**
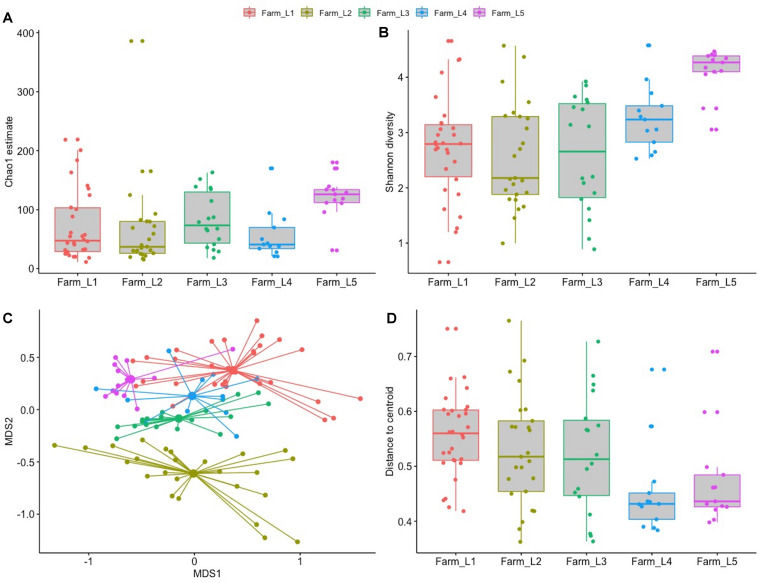
Alpha and beta diversity of the bulk tank milk microbiota of five farms sampled over a period of several months. **(A)** Chao1 richness estimation grouped by farm. **(B)** Shannon diversity grouped by farm. **(C)** NMDS plot of the bulk tank microbiota obtained using the Bray–Curtis dissimilarity matrix. **(D)** Beta dispersion of homogeneity grouped by farm.

**FIGURE 7:**
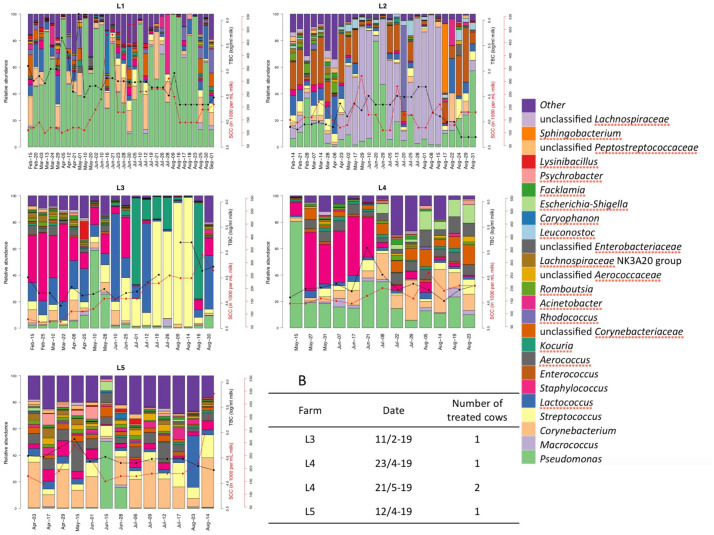
Relative abundance of the bulk tank milk microbiota of five farms (L1–L5) in the period February–August 2019. Somatic cell count (red lines) and total bacterial count (black lines) are reported for the milk delivered from the farms in the same period of the microbiota analysis. B: list of clinical mastitis cases reported by the five farms during the period of sampling and the number of cows treated. Somatic cell count, bacterial count and clinical mastitis reports were obtained from the Norwegian Dairy Herd Recording System.

A total of 5.4 million high quality sequences were obtained from the 99 samples with an average of 5,446 per sample (median 3,987). The sequences were divided into 1,186 unique SVs and taxonomically classified in 31 orders and 82 families. *Pseudomonas* was the most abundant genus (27.3%) within the microbiota, followed by *Macrococcus* (9.3%), *Corynebacterium* (8.2%), and *Streptococcus* (7.8%). Microbial richness and bacterial diversity were significantly different between the five farms with farm L5 having the highest estimate of bacterial taxa and the highest diversity calculated using the Shannon diversity index (Figure 6). This was also the farm with the highest geometric average for bulk milk SCC in 2019 (Figure 7 and Supplementary Table 1). Higher distribution of the Shannon diversity was detected for farms L1, L2, and L3 despite a similar microbial richness. Composition dissimilarities between the raw milk microbiota were calculated using the Bray–Curtis dissimilarity matrix. Distinct microbial compositions were detected between the farms (Figure 6). Both the microbial composition and the distance to centroid were significantly different between farms (adonis *P*-value < 0.001 and Kruskal–Wallis *P*-value 0.003, respectively) indicating a different microbial composition between the farms.

The farm L1 microbiota showed the highest abundance of *Pseudomonas* with a mean of 55% of the reads among all the 34 samples from this farm compared to the other farms (range 5.2–22.5%, Figure 7). This farm had the highest TBC among the five farms and also the highest SCC in the bulk tank milk. The farm had an average size of 12 cows in 2019 (Supplementary Table 1). During interviews, the farmer reported problems with the cleaning of the milking machine in the summer months of 2019. The genus *Macrococcus* was detected in high abundance in one farm (L2) (mean value 33.7%), while in the other farms this genus was only detected with an average lower than 2.2% of the reads. Samples from farm L2 with higher levels of *Macrococcus* also had higher TBC (Figure 7). Conversely, samples with lower levels of *Macrococcus* had reduced TBC.

Milk microbiota from farm L3 and L4 showed a temporary increase in the presence of bacterial taxa known to be associated with mastitis (*Staphylococcus* and *Streptococcus*). Milk samples from farm L3 contained a high amount of *Staphylococcus* at the beginning of the sampling period, and also reported a case of clinical mastitis in the same period. *Staphylococcus* was detected in high abundance for a long period (2 months) in the milk collected from this farm. Both SCC and TBC did not seem to be affected by the presence of *Staphylococcus.* The same farm did, however, have an increase in both SCC and TBC of bulk milk that coincided with an increased abundance of the genus *Streptococcus* and *Kocuria* during the summer months. Subsequently a decrease in TBC was observed in the same period where a lower abundance of *Streptococcus* was detected. Farm L4 reported two cases of clinical mastitis in lactating cows. One before the start of the sampling period and one between the first and second samplings. A relative increase in the abundance of *Staphylococcus* was observed between these two samplings, and this genus was detected in the milk over a period of 1 month.

Samples obtained from farm L5 showed the lowest beta dispersion and the highest similarity in microbial composition. The exception was one sample collected in June where an increase in abundance of *Pseudomonas* was detected. This farm reported a clinical mastitis case during the period of sampling but no changes in microbiota composition were detected.

## Discussion

In this study, we employed high throughput sequencing to uncover short-term and long-term longitudinal changes in bulk milk microbiota, and to characterize the diversity of microbes found in milk between farms. In addition, we used a questionnaire to farmers, the NDHRS and weather data to identify possible causes of microbial shifts in bulk tank milk.

While short term changes over weeks might reflect the seasonal effect and animal health (mastitis), the study of long-term changes over years provides a broader insight into the overall composition over time. To this end, we decided to repeat sample collection and analyses of bulk tank milk samples from the 37 farms sampled in 2017 using the same procedure (Skeie et al., 2019). Interestingly, we found that the microbial composition of farm milk divided in two geographical areas was significantly different in both alpha and beta diversity between the years. In particular, the presence of spoilage bacteria, such as *Pseudomonas* and *Bacillus* was significantly higher in the milk samples included in the latter sampling compared to the samples from the former study. This might pose a problem for the dairy industry as the presence of these two genera impact the milk quality negatively both before and after processing (Gopal et al., 2015; Martins et al., 2015). On the other hand, the abundance of *Lactococcus* was reduced in the samples from 2019 compared to 2017. This microbiota difference between the two years (2017 and 2019) might be attributed to several factors. As the milk samples were collected in the same manner, from the same farms and in the same period of the year, our experimental design allowed us to look beyond variation connected to season, hygiene practises, storage of milk, and sampling routines. The collection of metadata from each farm, also allowed us to identify changes on the farms between the two years. For example, the number of farms with AMS and farm size (number of animals) increased in area A. In addition, substantial differences were recorded in meteorological data in the summers before the sampling which likely affected feed management and feed quality. The increase in number of AMS farms might explain the higher richness and TBC found in the 2019 versus 2017 samples from area A. AMS have previously been associated with higher TBC due to poor cleaning of the teat. The teat microbiota has been hypothesized as the major source of bacteria in milk (Hogenboom et al., 2019). In this study, we detected that several taxa, such as *Corynebacterium*, *Ruminococcus*, *Aerococcus*, and *Facklamia*, were significantly more abundant in AMS compared to TSP and parlor milking system and these taxa were previously found in the teat microbiome (Falentin et al., 2016). However, the milking system cannot explain the differences in microbial diversity between the years as farms in area B did not change the milking systems. Bacterial taxa, not associated with the milking system, contributed to the diversity between the two years. In particular, environmental taxa such as *Pseudomonas*, *Bacillus*, *Paenibacillus*, and *Stenotrophomonas*, increased in abundance (mainly in area B) in samples from 2019, while *Lactococcus* decreased in abundance between the 2017 and 2019. In a comparative study of the bulk tank milk microbiota sampled in different countries, Parente et al. (2020) showed that the 2017 milk samples contained a higher amount of *Pseudomonas* and *Bacillus* compared to bulk tank milk from other countries. In this study, we confirmed the same finding and showed an increase of these spoilage bacteria in the bulk tank milk. The environmental taxa that (most probably) explained this microbial diversity between the years might originate from several sources. Feed and feeding regime has been shown to impact the bacterial community in milk (Fretin et al., 2018). While we did not have data on the quality and type of forage used during the two periods of samplings, results from the questionnaire confirmed that the farms used more concentrate and increased the amount of purchased roughage, including imported roughage, due to a drought in the summer of 2018, which might have affected the different microbiota.

In addition to the year-to-year variation, the geographical area might impact the microbiota. Previously we showed that the farm milk microbiota contains specific signatures linked to the geographical area (e.g., *Paenibacillus* was more abundant in area B and *Bacillus* in area A). After two years these specific signatures were still present, indicating that geographical location may have an impact on the microbiota found in milk. Differences in farm systems and herd size were detected between the two geographical areas, which might also contribute to differences in bulk milk microbiota. The possibility of contamination, which has been a challenge in milk microbiota studies (Taponen et al., 2019), cannot be firmly ruled out. In both 2017 and 2019, milk from farms in area A was collected by the same truck, while the milk from farms in area B were collected by three different trucks. The automatic sampling procedure is not completely sterile, as the device is cleaned with pressurized air between the farms. Cross contamination between farms has been reported using this sampling procedure (Andersen et al., 2003). To assess possible patterns of contamination, the abundance of the main genera was visually inspected between samples in the same order as the milk was collected from the farms. However, no connection between the order of milk collection and the microbiota composition was observed.

Microbiota analysis is biased by the analysis of relative abundance in the sample, as the change in abundance of one taxon is constrained to the increase or decrease of other taxa. By using absolute value we were able to correlate changes in bacterial counts with the taxa that increased or decreased in abundance. In this study, however, we did not observe changes in abundance for taxa such as *Streptococcus* and *Corynebacterium*, suggesting that the different composition between the *Lactococcus* and *Pseudomonas/Bacillus* taxa was actually related to their real abundance in the samples.

In addition to long-term microbiota changes we wanted to evaluate the short-term changes that might occur within the same farm. Alpha and beta diversity indexes showed that milk microbiota was farm-dependent, affirming our previous observations that each farm contains its own specific microbiota (Skeie et al., 2019). This indicates that farm environments and management play an important role on the microbial quality of milk delivered to the dairy industry (Doyle et al., 2017; Parente et al., 2020). The shorter longitudinal study showed that the microbiota is subject to continuous disturbance and fluctuation of abundance of some selected taxa. The major disturbance factor detected was the presence of mastitis-associated bacterial genera (*Staphylococcus*, *Streptococcus*, and *Macrococcus*) in addition to *Enterococcus*. These genera may originate from contamination from the teat or environment, but their increase in abundance was correlated with mastitis records (for *Staphylococcus*) or milk quality parameters (TBC and SCC for *Streptococcus* and *Macrococcus*). These observations indicate that udder health influences the abundance of these genera in bulk milk.

The longitudinal set up of the experiment allowed us to also evaluate how long the presence of the potential pathogens persisted in the bulk tank milk. *Staphylococcus* was detected in high abundance on two occasions in two different farms for a period of 1–2 months. In both cases, the clinical mastitis reports occurred with an increase of *Staphylococcus* abundance detected in the bulk tank milk. The *Staphylococcus* genus includes major udder pathogens like *Staphylococcus aureus*, known to cause chronic udder infections with a potential for contagious spread in the herd (Ruegg, 2017; Rainard et al., 2018). The staphylococci are a heterogenous group consisting of species adapted to the skin, the udder or the environment. The coincidence of clinical mastitis treatments and increase in staphylococcal abundance in this study could indicate that other cows than the ones registered or treated are subclinically infected. The sudden disappearance of staphylococci might be due to infected udders being dried off, infected cows being culled or that their milk is kept out of the bulk tank. As we did not investigate the infection status on cow level in this study, this information was not available.

The abundance of staphylococcal species was not associated with high TBC in this study, which is in agreement with other studies (Katholm et al., 2012; Rodrigues et al., 2017). The presence of *Macrococcus* was connected to one particular farm and a high abundance of this genus in the bulk tank milk was detected over almost 5 months. This genus is also a commensal of the animal skin and is found in milk and dairy products. *Macrococcus* is uncommon as a cause of bovine mastitis in Norway (Østerås, 2019), although elsewhere some species within the genus are increasingly recognized as udder pathogens (Schwendener et al., 2017). Farm L2, where a high abundance of *Macrococcus* was associated with high TBC and SCC was the only organic farm in the study.

Species belonging to the *Streptococcus* genus, such as *Strep. dysgalactiae*, *Strep. uberis*, and *Strep. agalactiae* are well known for their role in bovine mastitis. A substantial influence of the udder specific streptococci on the TBC is described in several studies (Katholm et al., 2012; Rodrigues et al., 2017). Though environmental streptococci contribute to the bulk microbiota, the samples with high streptococcal abundance in this study were associated with an increase of SCC and TBC in the milk from the same period, indicating that the origin of these streptococci may have been infected udders. Streptococci from infected quarters may be shed in higher numbers compared to staphylococcal species (Schukken et al., 2011).

In addition to short-term microbial changes due to an increase in abundance of some genera, we detected some bacterial taxa which are commonly present in all the milk samples collected from all the farms in both studies. This was the case for the genera *Pseudomonas*, *Corynebacterium*, *Lactococcus*, and *Aerococcus.* The abundance of these genera in the milk microbiota was clearly farm-dependent and their relative contribution to the total microbiota was also dependent on the abundance of other genera. The persistence of these genera over the entire period of sampling indicates their ability to contaminate the milk from different sources such as the farm environment or the animal. *Pseudomonas* is a well-known environmental bacteria which is frequently found in bulk tank milk and continuously poses a threat to the milk quality during storage temperatures (Zhang et al., 2020). *Corynebacterium* is a genus commonly detected in raw milk and contains species of veterinary importance due to their ability to colonize the teat skin and udder. This genus has been shown to increase the somatic cells counts and induce mastitis (Goncalves et al., 2016). *Lactococcus* is another genus commonly found in raw milk and is widely used for the production of fermented dairy products. The presence of this genus in all the milk samples and over an extended period, also indicated its ability to thrive in the farm environment as well as its ability to adapt to niches on the bovine skin and in the udder as previously reported (Werner et al., 2014).

## Conclusion

The present study elucidated the temporal changes that occurred in the milk microbiota and possible causes for these changes. We demonstrated that short-term longitudinal changes in microbial quality of the bulk tank milk within the same farm are mostly driven by mastitis-related genera (*Staphylococcus and Streptococcus*) while a persistent microbiota is found over time in the milk. Major shifts over time in milk microbiota were not correlated with milking system, number of cows or quality of the milk. One major difference between the two years of sampling was the weather during the harvesting seasons. This might have contributed to the shift in composition of the bulk tank milk microbiota. While we were not able to detect the exact causes of these changes, this study sets the scene for future investigations to determine factors of great importance for bulk milk microbiota at farm level. Furthermore, the data presented here indicate that in future regular assessment of farm bulk milk microbiota might be important to help to inform strategies to improve the microbial quality of milk.

## Data Availability Statement

The datasets presented in this study can be found in online repositories. The names of the repository/repositories and accession number(s) can be found below: https://www.ebi.ac.uk/ena, PRJEB39376.

## Author Contributions

DP designed and performed the microbiota experiments, performed the data analysis, and wrote the manuscript. MS contributed with the collection of data, analysis of the farm data, and writing of the manuscript. AA and AB contributed to the sample collection and microbiota analysis. HJ contributed with design and writing of the manuscript. SS designed and supervised the experiments and contributed to writing the manuscript. All authors contributed to the article and approved the submitted version.

## Conflict of Interest

The authors declare that the research was conducted in the absence of any commercial or financial relationships that could be construed as a potential conflict of interest.

## Supplementary Material

Supplementary Figure 1 Multivariate homogeneity of group dispersion grouped by area of sampling and year.

Supplementary Figure 2 Relative distribution of the 25 most abundant genera detected in raw milk samples. Each bar represents a sample of milk and each farm was sampled three times. A: Farm 1–19 collected in area A; B: farm 1–18 collected in area B. 2017: samples collected in the year 2017 and 2019: samples collected in 2019. Data from 2017 were previously published in Skeie et al. (2019).

Supplementary Table 1 Metadata from the five farms included in the study.

Supplementary Table 2 Questions and results used in the questionnaire submitted to the 37 farmers (33 replied) regarding the feed management practises that occurred between the 2019 and 2017 due to the 2018 summer drought.

## References

[B1] Andersen H. J. Pedersen L. H. Aarestrup F. M. Chriel M. (2003). Evaluation of the surveillance program of *Streptococcus agalactiae* in Danish dairy herds. *J. Dairy Sci.* 86 1233–1239. 10.3168/jds.S0022-0302(03)73707-2 12741548

[B2] Callahan B. J. McMurdie P. J. Rosen M. J. Han A. W. Johnson A. J. A. Holmes S. P. (2016). DADA2: High-resolution sample inference from Illumina amplicon data. *Nat. Methods* 13 581–583. 10.1038/NMETH.3869 27214047 PMC4927377

[B3] Doyle C. J. Gleeson D. O’Toole P. W. Cotter P. D. (2017). Impacts of seasonal housing and teat preparation on raw milk microbiota: a high-throughput sequencing study. *Appl. Environ. Microb.* 83:e02694-16. 10.1128/AEM.02694-16 27815277 PMC5203630

[B4] Elmoslemany A. M. Keefe G. P. Dohoo I. R. Wichtel J. J. Stryhn H. Dingwell R. T. (2010). The association between bulk tank milk analysis for raw milk quality and on-farm management practices. *Prev. Vet. Med.* 95 32–40. 10.1016/j.prevetmed.2010.03.007 20381889

[B5] Falentin H. Rault L. Nicolas A. Bouchard D. S. Lassalas J. Lamberton P. (2016). Bovine teat microbiome analysis revealed reduced alpha diversity and significant changes in taxonomic profiles in quarters with a history of mastitis. *Front. Microbiol* 7:480. 10.3389/fmicb.2016.00480 27242672 PMC4876361

[B6] Fretin M. Martin B. Rifa E. Isabelle V. M. Pomies D. Ferlay A. (2018). Bacterial community assembly from cow teat skin to ripened cheeses is influenced by grazing systems. *Sci. Rep.* 8:200. 10.1038/s41598-017-18447-y 29317671 PMC5760519

[B7] Goncalves J. L. Tomazi T. Barreiro J. R. Beuron D. C. Arcari M. A. Lee S. H. I. (2016). Effects of bovine subclinical mastitis caused by *Corynebacterium* spp. on somatic cell count, milk yield and composition by comparing contralateral quarters. *Vet. J.* 209 87–92. 10.1016/j.tvjl.2015.08.009 26831159

[B8] Gopal N. Hill C. Ross P. R. Beresford T. P. Fenelon M. A. Cotter P. D. (2015). The prevalence and control of *Bacillus* and related spore-forming bacteria in the dairy industry. *Front. Microbiol.* 6:01418. 10.3389/fmicb.2015.01418 26733963 PMC4685140

[B9] Hogenboom J. A. Pellegrino L. Sandrucci A. Rosi V. D’Incecco P. (2019). Invited review: Hygienic quality, composition, and technological performance of raw milk obtained by robotic milking of cows. *J. Dairy Sci.* 102 7640–7654. 10.3168/jds.2018-16013 31255272

[B10] Katholm J. Bennedsgaard T. W. Koskinen M. T. Rattenborg E. (2012). Quality of bulk tank milk samples from Danish dairy herds based on real-time polymerase chain reaction identification of mastitis pathogens. *J. Dairy Sci.* 95 5702–5708. 10.3168/jds.2011-5307 22921631

[B11] Lin H. Das Peddada S. (2020). Analysis of compositions of microbiomes with bias correction. *Nat. Commun.* 11:3514. 10.1038/s41467-020-17041-7 32665548 PMC7360769

[B12] Martins M. L. Pinto U. M. Riedel K. Vanetti M. C. D. (2015). Milk-deteriorating exoenzymes from *Pseudomonas fluorescens* 041 isolated from refrigerated raw milk. *Braz. J. Microbiol.* 46 207–217. 10.1590/S1517-838246120130859 26221110 PMC4512081

[B13] Mateos A. Guyard-Nicodeme M. Bagliniere F. Jardin J. Gaucheron F. Dary A. (2015). Proteolysis of milk proteins by AprX, an extracellular protease identified in *Pseudomonas* LBSA1 isolated from bulk raw milk, and implications for the stability of UHT milk. *Int. Dairy J.* 49 78–88. 10.1016/j.idairyj.2015.04.008

[B14] Norsk Klimaservicesenter (2019). Available online at: https://seklima.met.no/observations/ (accessed November, 2020).

[B15] Oksanen J. Blanchet F. G. Friendly F. Kindt R. Legendre P. McGlinn D. (2017). *vegan: Community Ecology Package.* R package version 2.4-2. Available online at: https://CRAN.R-project.org/package=vegan (accessed March, 2020).

[B16] Østerås O. (2019). *Helsekortordningen, Storfe 2018-Statistikksamling.* Available online at: https://www.animalia.no/no/Dyr/storfe/helsekort-og-beskrivelse-av-helsekortordningen/ (accessed May, 2020).

[B17] Parente E. Ricciardi A. Zotta T. (2020). The microbiota of dairy milk: a review. *Int. Dairy J* 107:104714. 10.1016/j.idairyj.2020.104714

[B18] Paulson J. N. Stine O. C. Bravo H. C. Pop M. (2013). Differential abundance analysis for microbial marker-gene surveys. *Nat. Methods* 10 1200–1202. 10.1038/NMETH.2658 24076764 PMC4010126

[B19] Porcellato D. Skeie S. B. (2016). Bacterial dynamics and functional analysis of microbial metagenomes during ripening of Dutch-type cheese. *Int. Dairy J.* 61 182–188. 10.1016/j.idairyj.2016.05.005

[B20] Quast C. Pruesse E. Yilmaz P. Gerken J. Schweer T. Yarza P. (2013). The SILVA ribosomal RNA gene database project: improved data processing and web-based tools. *Nucleic Acids Res.* 41 D590–D596. 10.1093/nar/gks1219 23193283 PMC3531112

[B21] R Core Team (2017). *R: A Language and Environment for Statistical Computing.* Vienna: R Foundation for Statistical Computing.

[B22] Rainard P. Foucras G. Fitzgerald J. R. Watts J. L. Koop G. Middleton J. R. (2018). Knowledge gaps and research priorities in *Staphylococcus aureus* mastitis control. *Transbound Emerg. Dis.* 65 149–165. 10.1111/tbed.12698 28984427

[B23] Rodrigues M. X. Lima S. F. Canniatti-Brazaca S. G. Bicalho R. C. (2017). The microbiome of bulk tank milk: characterization and associations with somatic cell count and bacterial count. *J. Dairy Sci.* 100 2536–2552. 10.3168/jds.2016-11540 28189327

[B24] Ruegg P. L. (2017). A 100-year review: mastitis detection, management, and prevention. *J. Dairy Sci.* 100 10381–10397. 10.3168/jds.2017-13023 29153171

[B25] Schukken Y. H. Gunther J. Fitzpatrick J. Fontaine M. C. Goetze L. Holst O. (2011). Host-response patterns of intramammary infections in dairy cows. *Vet. Immunol. Immunop.* 144 270–289. 10.1016/j.vetimm.2011.08.022 21955443

[B26] Schwendener S. Cotting K. Perreten V. (2017). Novel methicillin resistance gene mecD in clinical *Macrococcus caseolyticus* strains from bovine and canine sources. *Sci. Rep.* 7:43797. 10.1038/sre43797 PMC5341023 28272476

[B27] Skeie S. B. Haland M. Thorsen I. M. Narvhus J. Porcellato D. (2019). Bulk tank raw milk microbiota differs within and between farms: a moving goalpost challenging quality control. *J. Dairy Sci.* 102 1959–1971. 10.3168/jds.2017-14083 30639011

[B28] Stoddard S. F. Smith B. J. Hein R. Roller B. R. K. Schmidt T. M. (2015). rrnDB: improved tools for interpreting rRNA gene abundance in bacteria and archaea and a new foundation for future development. *Nucleic Acids Res.* 43 D593–D598. 10.1093/nar/gku1201 25414355 PMC4383981

[B29] Taponen S. McGuinness D. Hiitio H. Simojoki H. Zadoks R. Pyorala S. (2019). Bovine milk microbiome: a more complex issue than expected. *Vet. Res* 50:44. 10.1186/s13567-019-0662-y PMC6555717 31171032

[B30] TINE Rådgivingog Medlem (2019). Available online at: https://medlem.tine.no/aktuelt/nyheter/husdyrkontrollen/_attachment/499536?_ts=171c62ad785 (accessed November, 2020).

[B31] Werner B. Moroni P. Gioia G. Lavin-Alconero L. Yousaf A. Charter M. E. (2014). Short communication: genotypic and phenotypic identification of environmental streptococci and association of *Lactococcus lactis* ssp. *lactis* with intramammary infections among different dairy farms. *J. Dairy Sci.* 97 6964–6969. 10.3168/jds.2014-8314 25242419

[B32] Wright E. S. (2015). DECIPHER: harnessing local sequence context to improve protein multiple sequence alignment. *Bmc Bioinformatics* 16:322. 10.1186/s12859-015-0749-z PMC4595117 26445311

[B33] Zhang D. Li S. Palmer J. Teh K. H. Leow S. Flint S. (2020). The relationship between numbers of *Pseudomonas* bacteria in milk used to manufacture UHT milk and the effect on product quality. *Int. Dairy J* 105:104687. 10.1016/j.idairyj.2020.104687

