# Supplementary figures and images for: Longitudinal Study of the Bulk Tank Milk Microbiota Reveals Major Temporal Shifts in Composition
Source: Front Microbiol. 2021 Feb 23;12:616429. doi: 10.3389/fmicb.2021.616429 (PMC7940241; doi:10.3389/fmicb.2021.616429)

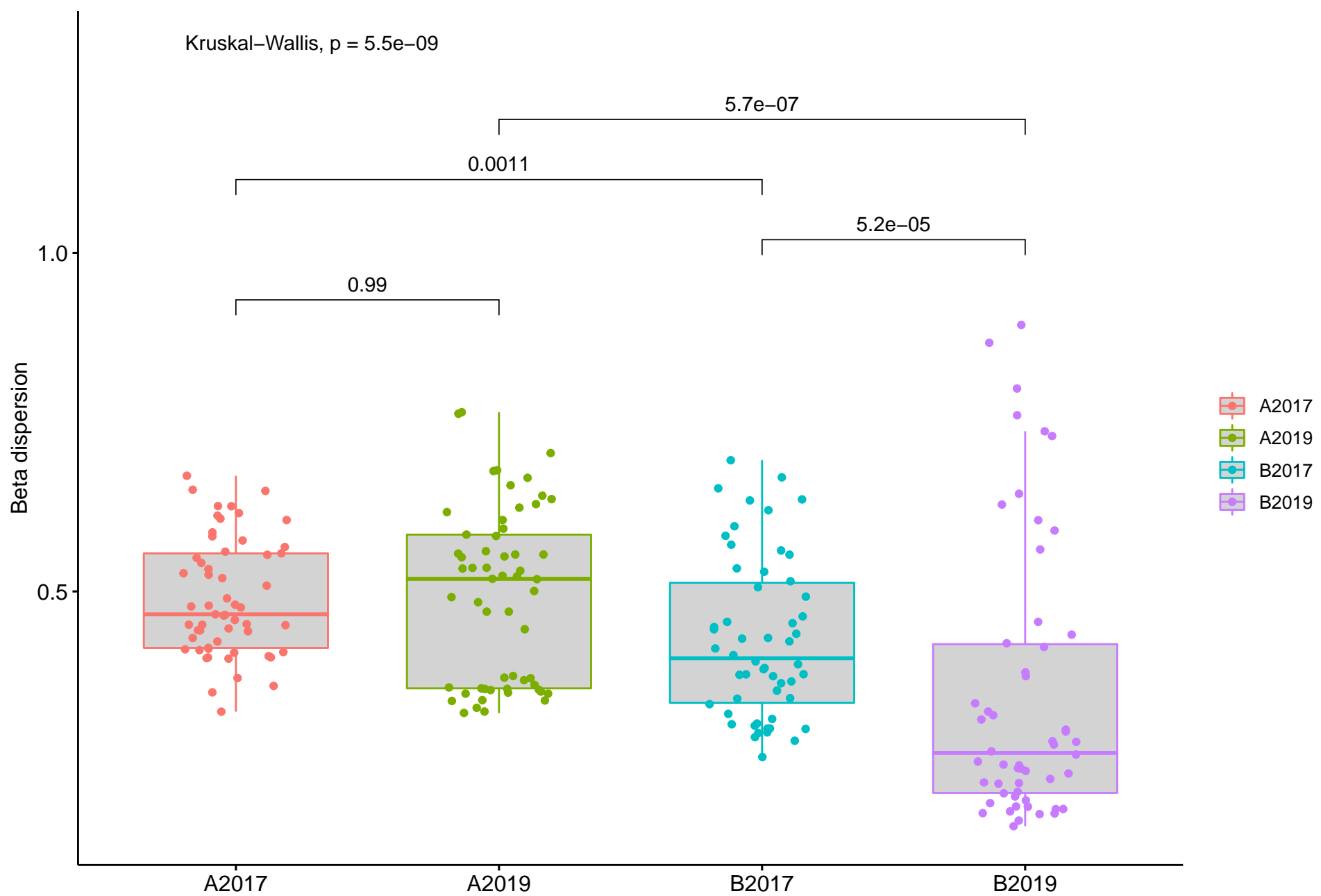

Supplement: Supplementary Figure 1 — Multivariate homogeneity of group dispersion grouped by area of sampling and year. [file Data_Sheet_1.PDF]

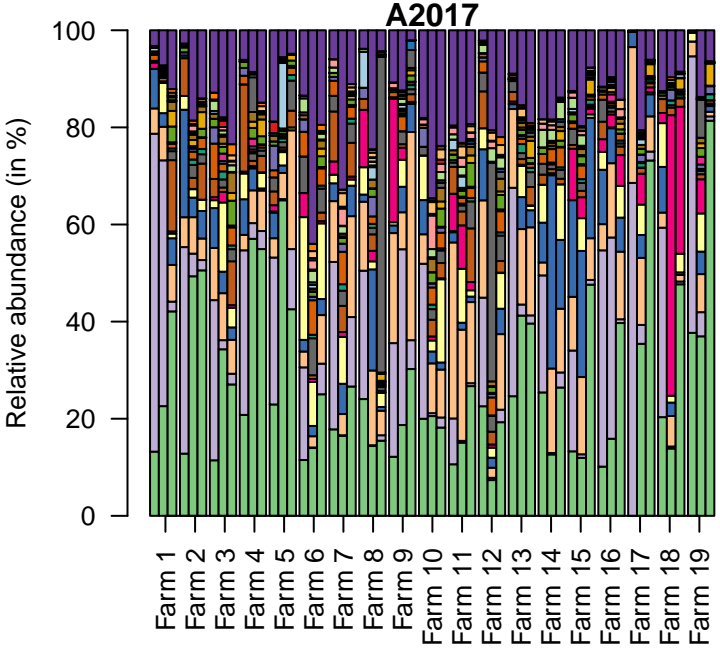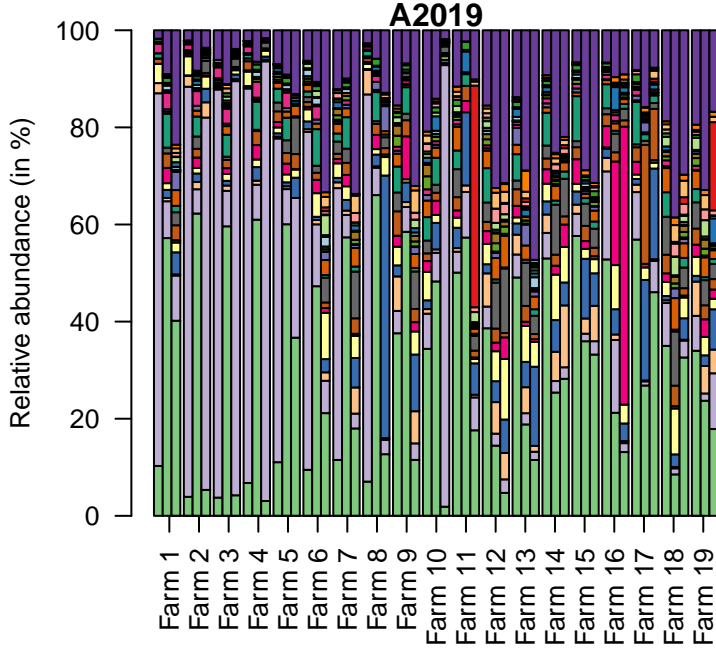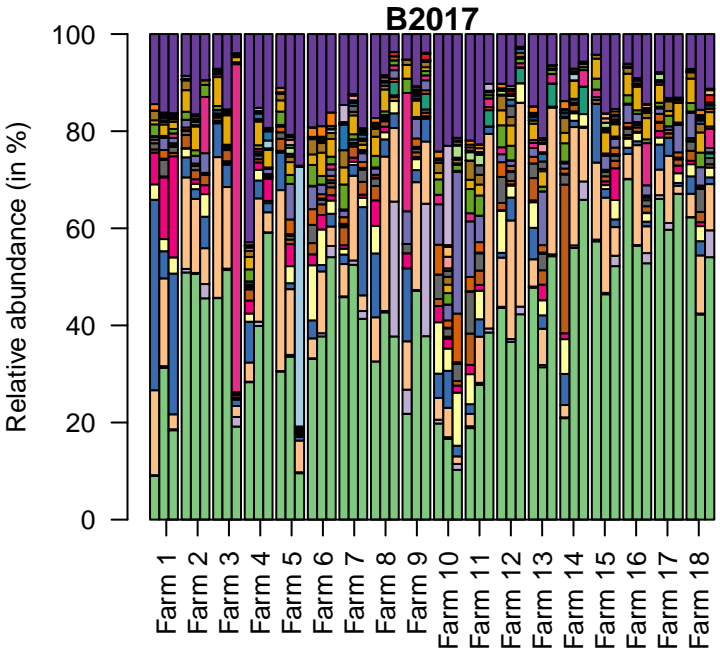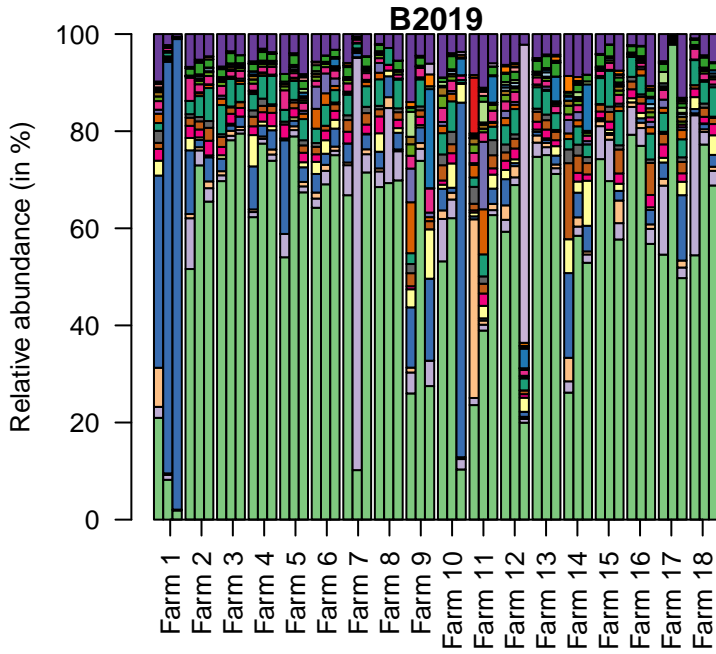

Supplement: Supplementary Figure 2 — Relative distribution of the 25 most abundant genera detected in raw milk samples. Each bar represents a sample of milk and each farm was sampled three times. A: Farm 1–19 collected in area A; B: farm 1–18 collected in area B. 2017: samples collected in the year 2017 and 2019: samples collected in 2019. Data from 2017 were previously published in Skeie et al. (2019). [file Data_Sheet_2.PDF]
