# Supplementary material for: Longitudinal Study of the Bulk Tank Milk Microbiota Reveals Major Temporal Shifts in Composition
Source: Front Microbiol. 2021 Feb 23;12:616429. doi: 10.3389/fmicb.2021.616429 (PMC7940241; doi:10.3389/fmicb.2021.616429)
Supplement: Supplementary Table 1 — Metadata from the five farms included in the study. [file Table_1.DOCX]

**Table S1. Metadata from the five farms included in the study.**

| **Farm** | **Farm type** | **Milking system** | **Cow years**  **(2019)** | **Kg milk/cow (ECM)** | **Somatic cell count **** | **Clinical mastitis treatments and date of treatments** | **Dry cow therapy during sampling period** |
| --- | --- | --- | --- | --- | --- | --- | --- |
| L1 | Conventional | Milking parlour | 12 | 6618 | 160 | 0 | 0 |
| L2 | Organic | AMS* | 41 | 8669 | 132 | 0 | 0 |
| L3 | Conventional | AMS | 50 | 9657 | 126 | 1 (11/2-19) | 0 |
| L4 | Conventional | AMS | 49 | 8714 | 161 | 3 (23/4-19, 21/5-19 (2 cows)) | 0 |
| L5 | Conventional | AMS | 34 | 8707 | 196 | 1 (12/4-19) | 0 |

***AMS: automatic milking system**

****geometric average for 2019 in 1000 per mL of milk**
